# Combining visual motion and luminance features to enhance the detection of small moving objects in a bioinspired model

**DOI:** 10.1371/journal.pcbi.1014036

**Published:** 2026-03-02

**Authors:** Shuai Li, Aike Guo, Yizheng Wang, Liang Li, Gang Wang, Zhihua Wu

**Affiliations:** 1 School of Life Sciences, Shanghai University, Shanghai, China; 2 International Academic Center of Complex Systems, Advanced Institute of Natural Sciences, Beijing Normal University at Zhuhai, Zhuhai, Guangdong, China; 3 Brain Research Center, Beijing Institute of Basic Medical Sciences, Beijing, China; Soochow University, CHINA

## Abstract

Flying insects demonstrate exceptional proficiency in detecting and pursuing conspecifics and prey within a cluttered environment, inspiring the development of computational models for small object detection. While existing bioinspired models are dedicated to resolving small moving instead of stationary object detection, few studies have systematically explored the role of visual motion in detection. Here, we developed a fly-inspired model on the basis of the hypothesis that combining visual motion features and luminance features is critical for small moving object detection. We thoroughly investigated the effect of feature combination under diverse stimulus conditions. Simulations indicated that the model exhibited hyperacute object detection, a capability not generally believed to emerge on the basis of motion detection. When tested with a moving background in realistic scenarios, the model demonstrated enhanced efficiency and robustness relative to models relying solely on luminance features. This enhancement was independent of whether visual motion was extracted by two- or three-arm motion detectors. The results suggested that small object detectors within the visual systems of flying insects could be optimally tuned to utilize the limited features inherent to tiny objects.

## Author summary

Flying insects are adept at detecting small moving objects on a daily basis under natural conditions, even if these objects are smaller than the spatial resolution of their compound eyes. In contrast, small object detection is still a challenge for artificial vision systems, such as those used for robots, even with the help of deep convolutional neural networks. The main challenge is that tiny objects have few visual features. Thus, it is valuable to draw inspiration from the neural algorithms of miniature insect brains, which resolve this issue by relying on the limited features of a tiny object. While a variety of neurons selectively responding to the movement of small objects have been identified in fruit flies, hoverflies, and dragonflies, little is known about whether the neurons specifically responsible for detecting visual motion are involved in small moving object detection. Systematic investigations of how visual motion affects tiny object detection are lacking in modeling studies. Simply put, the role of visual motion, i.e., a conspicuous but underemphasized feature in existing models, in the detection of small objects remains poorly understood. We address this question by developing a parsimonious model that correlates the visual motion of the object’s leading edge with the luminance of its trailing edge. The results show that properly combining visual motion and luminance features can ensure the detection of small moving objects without the need for additional complex mechanisms. An evaluation of real-world datasets of small objects, including drones in infrared videos, indicates that the model outperforms existing methods inspired by flying insects and thus demonstrates potential for application in artificial vision systems.

## 1. Introduction

The neurons that have been identified as being able to selectively respond to small objects in flying insects [1−4] serve as references for evaluating the neural computations underlying small moving object detection. Given the heterogeneity in response characteristics among these neurons, different hypotheses have been proposed [2,4,5−7]. The most extensively studied model, inspired by the physiological properties of “small target motion detector (STMD)” neurons identified in the lobula of the hoverfly and dragonfly [1,2], is the elementary STMD (ESTMD) [2,5,6]. Each ESTMD unit can be assumed to be able to detect a small dark object by exploiting the temporal correlation between two edge signals of the object that are sequentially received from a location in the visual field [5]. This process is realized by multiplying the properly delayed OFF channel’s signal with the ON channel’s signal; these signals are sequentially activated by the object’s leading and trailing edges at the same location.

While STMD neurons have not yet been found in *Drosophila*, types of lobula columnar (LC) cells including LC11 and LC18 in the *Drosophila* lobula have been shown to respond specifically and robustly to small objects [3,4,7]. Unlike STMD neurons, LC11 neurons have been shown to emerge without the need for interaction between the OFF and ON pathways; this phenomenon arises because of the spatial pooling of size-tuned, fast-adapting units [7]. LC18 neurons have been proposed to require linear rather than nonlinear interactions between slower and faster signals that are not only across but also within the ON and OFF channels, characterizing their different responses from those of STMD neurons [4].

Although being different in detail, the above models share a common hypothesis that small object detectors do not involve visual motion but instead rely only on luminance. Luminance is defined as the output intensity of photoreceptor transduction rather than the light intensity directly sampled by photoreceptors, which are simulated as pixel values of input images (Methods). The spatiotemporal cross-correlation of luminance signals offset in space and time causes the perception of visual motion, which is analyzed or computed with the pathway from the retina to T4/T5 cells in flies. The computation of visual motion was formulated by correlation-type elementary motion detectors (EMDs) more than half a century ago [8]. Individual EMDs are currently proposed to have three rather than two spatially separated inputs, as recent studies have revealed that T4/T5 cells detect visual motion in a manner that can be described by a three-arm detector model [9–15].

In a previous model [16], it was proposed that ESTMDs should receive visual motion input from EMDs to account for the direction selectivity of one type of STMD neuron [17,18]. Compared with nondirectionally selective ESTMDs [2,5,6], however, the cascaded EMD–ESTMD model [16] does not explain what advantages the direction selectivity of ESTMDs can bring to small object detection. Another type of model indicates that the temporal correlation between two ESTMDs, with a distance three times the spacing of the ESTMD array, is essential for the generation of ESTMD directional selectivity [19,20]. Since only motion information with a displacement not less than three times the spacing between adjacent ESTMDs is extracted, this type of model does not involve visual motion; visual motion can be elicited from the temporal correlation between two adjacent ommatidia or points anywhere in the visual field [21,22]. Therefore, whether visual motion is necessary for small moving object detection remains elusive. It is less clear whether the direction of visual motion is critical for detecting small objects.

In this study, we hypothesized that visual motion features are just as critical as luminance features for small moving object detection. By combining the two features, we developed a computational model and thoroughly investigated how feature combination contributes to small object detection. Simulations were performed under a variety of visual stimulus conditions, including tiny moving objects with size below the spatial resolution of the modeled eye, moving backgrounds, and real-world video sequences. The results indicate that the model guided by visual motion is much more efficient and robust than ESTMD-based models that rely on luminance alone. The conclusion is independent of whether visual motion is extracted by two- or three-arm detectors.

## 2. Results

### 2.1. Feature combination model

Inspired by the ESTMD model [2,5,6], our model treats small moving object detection as a multiplicative correlation between its trailing and delayed leading edges that have opposite polarities. Unlike the ESTMD model, which correlates luminance signals of two edges, our model proposes that correlating the visual motion features of the leading edge with the luminance features of the trailing edge at the same retinotopic location is critical for improving detection performance. Specifically, small, independently moving object detection is triggered by its visual motion feature. This property is in contrast to the case of small, static objects, which should be viewed as textural components of the background and may be detected by other nonmotion-dependent neural mechanisms [23].

**Fig 1:**
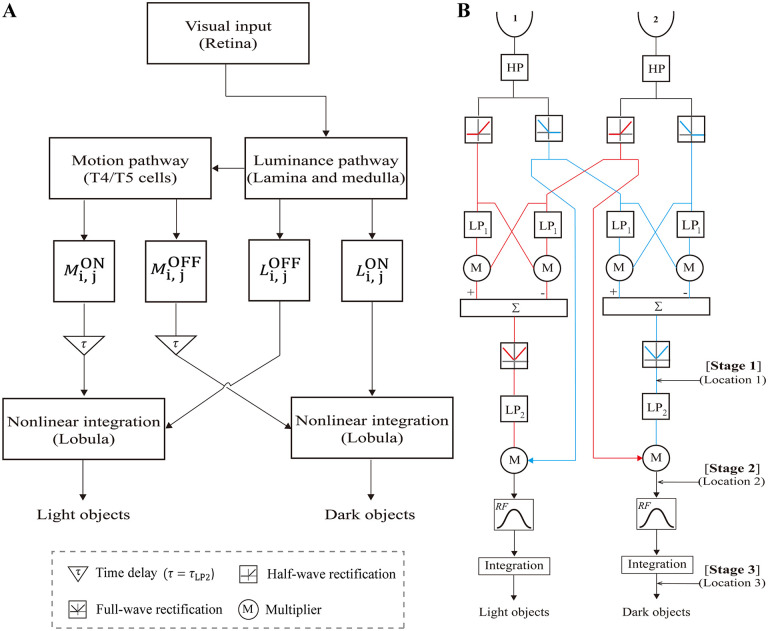
Feature combination model for small moving object detection. **(A)** Schematic of the model network. Each box expresses one visual calculation modle, whose neural relevance is marked in the parenthesis. See the text for the variables $M_{\text{i},\text{ j}}^{OFF}$, $L_{\text{i},\text{ j}}^{ON}$, etc. **(B)** Detailed model structure along either the horizontal or vertical axis at a pair of retinotopic locations. The input at each location is divided into parallel ON (red) and OFF (blue) channels. HP represents a first-order high-pass filter with a time constant of *τ*_HP_ = 30 ms. LP_1_ and LP_2_ represent first-order low-pass filters, with *τ*_LP1_ = 50 ms and *τ*_LP2_ = *τ* = 30 ms, respectively. Other symbolic representations are defined in the bottom left corner. EMD consists of two mirror- symmetric “half-correlators”, whose subtraction (signified by the symbol “∑”) yields visual motion signals with opposite signs along either the horizontal or vertical axis. Through full-wave rectifiers, the visual motion signals are transformed to positive values, producing the output of stage 1 at Location 1. Multiplying the delayed (signified by LP_2_) motion signals by the luminance signals of opposite polarity produces the output of stage 2 at Location 2. The output of stage 2 is then projected to the lobula module for spatiotemporal integration, producing the output of stage 3 at Location 3. *RF* represents the Gaussian receptive field of the lobula units, whose spatial size and standard deviation are 3Δφ×3Δφ and 0.5∆φ, respectively.

The model features two parallel pathways, each dedicated to the detection of small dark and light objects separately (Fig 1A). Both pathways share the same three-stage processing mechanism. The pathway for dark object detection is taken as an example. Stage 1 simulates the neural processing from the input to the sum of the T5 cell outputs and, thus, extracts OFF visual motion across the entire visual field regardless of direction. This process can be achieved by first applying a full-wave rectifier to the output of each EMD (Fig 1B) and then adding the rectified signals of EMD arrays retinotopically (Methods). Stage 2 nonlinearly combines undelayed luminance and delayed visual motion signals at the same retinotopic location with opposite polarity, which is hypothesized to occur in the lobula. This process is achieved by first applying first-order low-pass filtering to the output of stage 1 (denoted as *τ* in Fig 1A and LP2 in 1B) and then retinotopically multiplying the filtered visual motion signals (denoted as matrix $M_{\text{i},\text{ j}}^{OFF}$) by the luminance signals in the ON channel (denoted as matrix $L_{\text{i},\text{ j}}^{ON}$), yielding the output of stage 2 as $M_{\text{i},\text{ j}}^{OFF}\times L_{\text{i},\text{ j}}^{ON}$. The pathway for light object detection is the same as that for dark object detection, except for the polarity interchanged between the visual motion and luminance pathways.

In stage 3, spatiotemporal smoothing is performed on the input from stage 2. Stage 3 simulates the lobula columnar (LC) cells, which are hypothesized to have small receptive fields and be involved in small object detection. The LC units of the lobular module are modeled to encode the input with graded potentials (Eq (1)) [24,25], whose input conductance matrix $\;g_{\text{i},\text{ j}}$ (see Eq (2) in Methods) can be determined by a convolution as $g_{\text{i},\text{ j}}=\left(M_{\text{i},\text{ j}}^{OFF}\times L_{\text{i},\text{ j}}^{ON}\right)*RF$, where the kernel *RF* is the receptive field matrix of the lobula module. Unless otherwise specified, each receptive field of individual lobula units is set as a square area of 3Δφ×3Δφ, i.e., 3×3 EMDs, in which ∆φ is the spacing between adjacent EMDs. This value is equivalent to 18 pixels×18 pixels for the first stimulus type.

The model responses probed at Locations 1, 2, and 3 along the signal processing flow are the output of stages 1, 2, and 3, respectively (Fig 1B), which are denoted as the ml-SOD (Stage 1), the ml-SOD (Stage 2), and the ml-SOD (Stage 3) for convenience below, respectively.

In summary, we develop a fly-inspired model that can detect small, independently moving objects. The model multiplicatively combines the visual motion and luminance features of the object and is thus called the motion luminance small object detector (ml-SOD). The model proposes that the direction of visual motion is not necessary for small moving object detection. This property is in accordance with the fact that the LCs sensitive to small objects are motion-selective yet nondirectional [3,4].

### 2.2. Hyperacute detection and selectivity for object size and velocity

**Fig 2:**
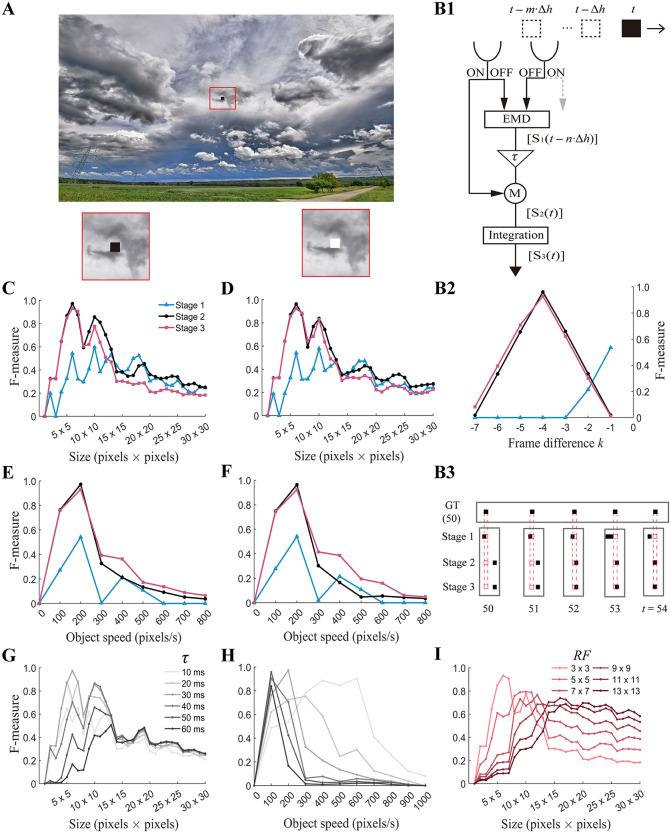
Model selectivity for object size and velocity. **(A)** An example of an input frame. A dark (0.0 luminance) (zoomed into the bottom left corner) or light (1.0 luminance) (zoomed into the bottom right corner) square object moves to the left at a constant velocity over a stationary image [26] background. Red boundaries outlining the magnified area are not present in the actual stimuli. **(B1)** Schematic of temporal alignment at a retinotopic location between an input object and the response at each model stage. The leading edge of the object captured by the left input channel at time (t−m·Δh) moves to the location marked by a black square at time *t*, during which the induced signal arrives at stage 1 (*S*_1_) and stages 2 (*S*_2_) and 3 (*S*_3_) at times (t−n·Δh) and *t*, respectively. Δ*h* is the sampling period of the input frames. **(B2)** F-measure of *S*_1_(*t*), *S*_2_(*t*), and *S*_3_(*t*) in B1 as a func*t*ion of the ground truth time. The negative integer *k* on the x-axis expresses that the F-measure is calculated using the ground truth *k* frames ago. **(B3)** Temporal alignment in B1 and B2 for *t* = 54 as an example. Horizontal box: ground truth (GT) corresponding to the output a*t* time ***t*.** Vertical boxes: model responses (stage names indicated to the left of each row) from *t* = 50 to *t* = 54 (bottom). Data across rows are from a patch of the model of *t*he same size 4Δφ×4Δφ, whose retinotopic correspondence relationships are exactly presented (red dashed boxes). **(C–F)** Tuning curves of the model. The F-measure of the signal probed at Locations 1, 2, and 3 (Fig 1B) varies with the size (C) or speed (E) of the dark moving object. D and F are the same as C and E, respectively, except that the dark object is replaced with a light object. **(G, H)** Size (G) and speed (H) tuning curves at stage 2 under different temporal delays *τ*. **(I)** Size tuning curve at stage 3 under different receptive field (*RF*) sizes of the lobula units. Unless specified in the plots, the object size, object speed, and *RF* size are set to 6 pixels×6 pixels, 200 pixels/sec, and 3Δφ×3Δφ, respectively. The color legend (C) is used throughout B2 and C–F.

We first test the ml-SOD model using artificial stimuli with adjustable object size and velocity parameters, and the results are evaluated by the F-measure (Methods) (Fig 2A). Given that the model depends on temporal delays produced by LP_1_ and LP_2_ (Fig 1B), temporally aligning the detected object with its true ground truth is critical for ensuring a fair evaluation of model performance, especially in the case of tiny object detection.

The detection of a small object at time *t* at a retinotopic location implies that the ground truth object passes the location several frames ago (denoted as (*t* ‒ *m*·Δ*h*)) and induces responses at stages 1, 2, and 3 at (*t* ‒ *n*·Δ*h*), *t*, and *t*, respectively, where Δ*h* is the time step of the frame input (Methods) and *m* and *n* are integers (Fig 2B1). Since stages 2 and 3 are after stage 1, we have *m* > *n*. To determine the values of *m* and *n*, the model performance with the object size and velocity set to intermediate values is repeatedly evaluated with a varied ground truth that systematically goes back in time (Fig 2B2). The results reveal that the ground truth that is aligned at stages 2 and 3 occurred four frames ago (i.e., *m* = 4) and that aligned at stage 1 occurred one frame ago (i.e., *n* = 3), as shown by the model responses during five consecutive frames in an example trial (Fig 2B3). The temporal alignment rule is applied to evaluate all the results with the artificial stimuli implemented throughout the study.

By fixing the object velocity between trials, simulations indicate the existence of a preferred object size at which the model exhibits optimal detection performance, regardless of whether the object is dark (Fig 2C) or light (Fig 2D). The size tuning curves are qualitatively in accordance with the size selectivity of types of LC cells to small objects in the *Drosophila* (e.g., Fig 2 in [4]). By fixing the object size while allowing its velocity to vary, the results reveal a preferred object velocity, regardless of whether the object is dark (Fig 2E) or light (Fig 2F). Stronger selectivity at stages 2 and 3 than at stage 1 demonstrates that the selectivity originates from the combination of visual motion and luminance features and, thus, should be modulated by parameters of feature combination. This prediction is confirmed by repeated simulations with systematically varied parameter values (Fig 2G‒2I). The preferred object size increases with increasing receptive field size of the lobula module (Fig 2I). The preferred object size and velocity increases and decreases, respectively, with the temporal delay *τ* *=* *τ*_LP2_ (Fig 2G, 2H). This result is in accordance with the fact that the total time for the object to pass through a retinotopic location, determined by its size and velocity, needs to match the temporal delay *τ* to achieve optimal detection.

**Fig 3:**
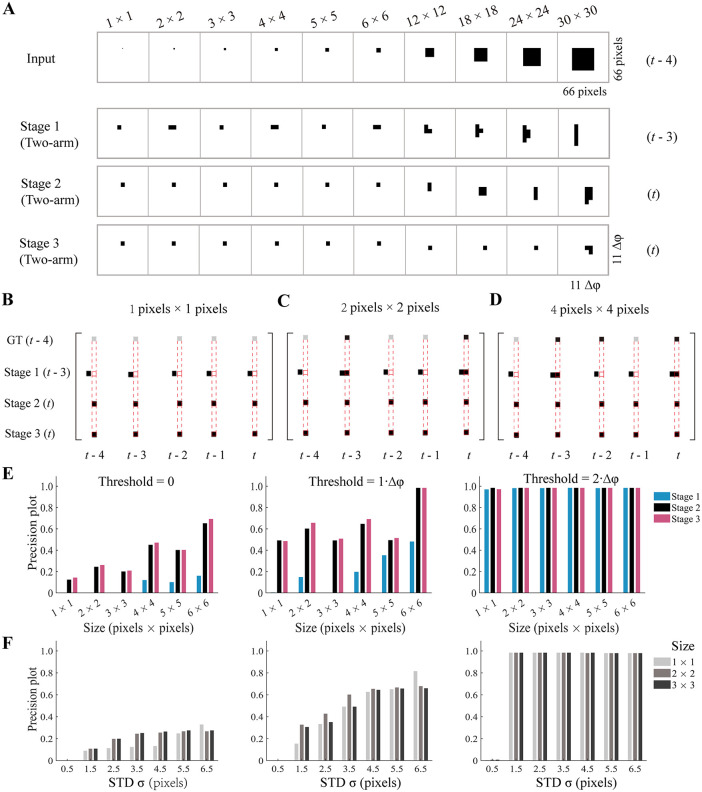
Hyperacute detection of the ml-SOD model. **(A)** Model responses around detected objects at a given instant (*t* = 55). 1st row: the input frames with different object sizes (top). 2nd‒4th rows: corresponding model responses with the frame (right) and stage number (left) indicated. Data across the four rows are from retinotopically corresponding patches of the model (marked in the rightmost panel of the 1st and 4th rows). **(B‒D)** Examples of ground truth and model responses with three object sizes (top). For each object size, data are presented for five consecutive frames (time marked at the bottom, *t* = 54). Each column displays the ground truth and model responses at each of the three stages, whose retinotopic relationships are exactly presented. The time varies between rows (indicated in the parentheses to the left of each row). The temporal alignment between the input and model responses is indicated by red dashed boxes. The gray squares in the 1st row indicate that the ground truth (GT) is lost because of the downsampling operation. **(E)** Precision plots for different distance thresholds (top). The data in A‒E are from the same simulations as those presented in Fig 2C. **(F)** Effect of the standard deviation of Gaussian blur on precision plots of model responses at stage 2. The panels sharing the same threshold are vertically aligned in E and **F.** Data for only three object sizes are shown.

To investigate the size selectivity in depth, we examine the temporally aligned response of a patch of the model with a size 11Δφ×11Δφ, corresponding to an input area of 66 pixels×66 pixels (Fig 3A). As derived from the model mechanism, size selectivity occurs mainly along the axis parallel, not orthogonal, to the motion of the object. Surprisingly, tiny objects, even those with the smallest size of 1 pixels×1 pixels, were detected at each of the three stages (Fig 3A). This finding is surprising, as the detection of moving objects smaller than a single facet of compound eyes is not generally believed to be based on comparisons of signals that are spatially separated [4]. However, it is theoretically feasible because a tiny object can be magnified via Gaussian blur preprocessing (Methods) and thus be detected.

The above observation (Fig 3A), however, contradicts the considerably low F-measures with tiny objects smaller than 6 pixels×6 pixels (Fig 2C and 2D). Further examination on a frame-by-frame basis ultimately reveals the underlying reason. Preprocessing to the ground truth of each input frame only involves downsampling but not Gaussian blur, which can cause loss of the ground truth of tiny objects smaller than the sampling interval ∆φ (Methods). The lose probability decreases with increasing object size (Fig 3B‒3D). Evaluating with losing ground truth results in an average F-measure close to 0 (Eq (3)), even if the tiny object is detected.

Given that the F-measure is no longer applicable for tiny objects smaller than Δφ×Δφ, we adopt a precision plot as an alternative (Methods). The metric needs a distance threshold. Since it takes *m* ∙ ∆*h* for an object moving at speed *V* to be detected at stages 2 and 3 (Fig 2B1–2B3), the detected object shifts by *V* ∙ *m* ∙ ∆*h* relative to the ground truth of the object location. By taking the median speed as *V* = 400 pixels/sec (see the speed range in Fig 2E and 2F) and the time as *m* ∙ ∆*h* = 40 ms, a distance threshold limit of 16 pixels, i.e., 2.7∆φ, can be estimated for the precision metric. A re-evaluation of the above results indicates that the precision of the detection of tiny objects notably increases with increasing distance threshold at all three stages (Fig 3E). Compared with stage 1, stages 2 and 3 showed much higher precision for low distance thresholds. When the distance threshold is increased to 2∆φ, which is still within the distance threshold limit, the precision metric reaches 1.0 at all three stages. The results confirm that the detection capability of the model exceeds the resolution limit ∆φ set by the modeled photoreceptor units.

Visual hyperacuity refers to a visual phenomenon in which the perception capability transcends sampling limits set by discrete photoreceptors in the retina [27]. Therefore, the model capability is determined as a type of visual hyperacuity and called as hyperacute object detection. In contrast to *Drosophila* hyperacute vision induced by the fast microsaccadic sampling of photoreceptors [28], hyperacute object detection is the synergistic result of two mechanisms. Specifically, weak visual motion signals detected due to retinal Gaussian blur are captured and amplified by the feature combination mechanism downstream of T4/T5 cells.

Finally, whether the application of Gaussian blur preprocessing necessarily guarantees hyperacute object detection remains unclear. By fixing the size of the Gaussian low-pass filter but allowing its standard deviation σ to vary, our repeated simulations indicate that the answer depends on the magnitude of σ (Fig 3F). In the case of a Gaussian low-pass filter with a size of 12×12 pixels, hyperacute object detection occurs only when the standard deviation σ is greater than 1.5 pixels.

Taken together, the ml-SOD model prefers small objects whose size *S* and velocity *V* approximately satisfy the relationship *S* = *V* x *τ*. Moreover, the model can detect tiny objects that are much smaller than its spatial resolution. The model predicts that hyperacute detection of subresolution moving objects exhibited in the low-resolution compound eye (e.g., [29]) may be achieved by a combination of visual motion and luminance features in the insect visual system.

### 2.3. Robust detection of moving high-contrast small objects against moving background

**Fig 4:**
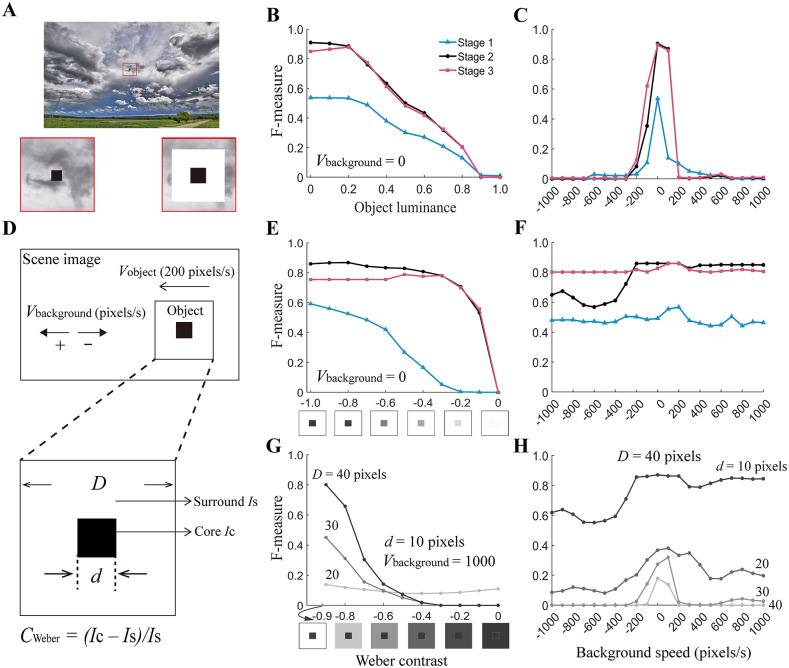
Robust detection of moving high-contrast objects against a moving background. **(A)** An example of a stimulus frame. The small dark object moved to the left (speed *V*_object_ = 200 pixels/sec) over an image [26] background (speed *V*_background_). Simulations, the results of which are shown in B and C, use the stimuli with an object of 6 pixels×6 pixels with luminance *I*_C_ (zoomed into the bottom left corner). Simulations, the results of which are shown in E‒F, use the stimuli with a high-contrast object made up of two overlapping squares (core: 10 pixels×10 pixels size and 0.0 luminance; border: 40 pixels×40 pixels size and 1.0 luminance) (zoomed into the bottom right corner). **(B)** Tuning curves of the model as a function of the *I*_C_ with *V*_background_ indicated. **(C)** Same as (B) except that *I*_C_ = 0.0 and the background is moving in the same (positive speed as indicated on the x-axis) or opposite (negative speed) direction relative to the object. **(D)** Schematic diagram of the high-contrast object shown in **A.** The core is d×d in size (luminance *I*_C_) and surrounded by a border of D×D (luminance *I*_S_). The local contrast of the object is evaluated by the Weber contrast (*C*_Weber_). **(E and F)** E and F are the same as B and C, respectively, except that the object is replaced with a high-contrast object. Weber contrast is controlled by fixing *I*_S_ = 1.0 and varying the *I*_C_ in the range of 0.0‒1.0 between trials **(E)**. We set *I*_S_ = 1.0 and *I*_C_ = 0.0 in **F. (G‒H)** Same as F except that the only data at model stage 2 are displayed. Note that we fix *V*_background_ = 1000 pixels/sec in **G.** Except for parameter values indicated in each panel, Weber contrast is controlled by fixing *I*_C_ = 0.1 but varying *I*_S_ in the range of 0.1‒1.0 between trials in G, and *I*_S_ = 1.0 and *I*_C_ = 0.0 in **H.** The color legend (B) is used throughout B, C, E and **F.** All the data are from the model pathway of dark object detection.

One challenge for object detection arises from the moving background [30]. This phenomenon is confirmed by our simulations under the condition of a set of stimuli, in which a small object moves at a constant velocity while the background speed is systematically changed across trials (Fig 4A and 4C). The poor performance with fast background speed occurs because the moving background textures similar to the object in size and velocity are captured by EMDs and mistakenly by the model mixed into the visual motion features of the real object.

Considering that visual motion has a square dependency on pattern contrast [31,32], we ask whether the model performance can be improved if the local contrast of the object is enhanced by surrounding it with the color white (the bottom right panel in Fig 4A and 4D). Repeated simulations reveal that the high-contrast object can be effectively detected at both stages 2 and 3 across the all background velocities tested (Fig 4F). This finding is in sharp contrast to the detection of the object with intact contrast, which is only detected when the background speed is slow enough (Fig 4C). The substantial effect of high object contrast is consistently reflected in object detection with a stationary background, as shown by the high F-measure across a wide range of Weber contrasts at stages 2 and 3 (Fig 4E). By contrast, in the case of the intact contrast object, the F-measure decreases almost linearly with object luminance (Fig 4B).

Notably, the substantial improvement above is not due to the enlargement of object sizes because the model prefers small but not large objects (Fig 2C and 2D). The size selectivity is further confirmed by simulations with the object border *D* being fixed and its core size *d* varying across trials (Fig 4H). By fixing the core size but allowing its Weber contrast to vary, the F-measure is showed to increase with the Weber contrast as long as the border size *D* is not too small, confirming the important role of the enhanced local contrast (Fig 4G).

In summary, the ml-SOD model is robust to interference arising from the moving background, as long as the contrast of small moving objects is adequately high.

### 2.4. Comparison with the ESTMD model

Given that both the ml-SOD and ESTMD models detect a small moving object by exploiting multiplicative interactions between its two edges, an obvious question to ask is whether the two models differ in terms of detection effectiveness.

**Fig 5:**
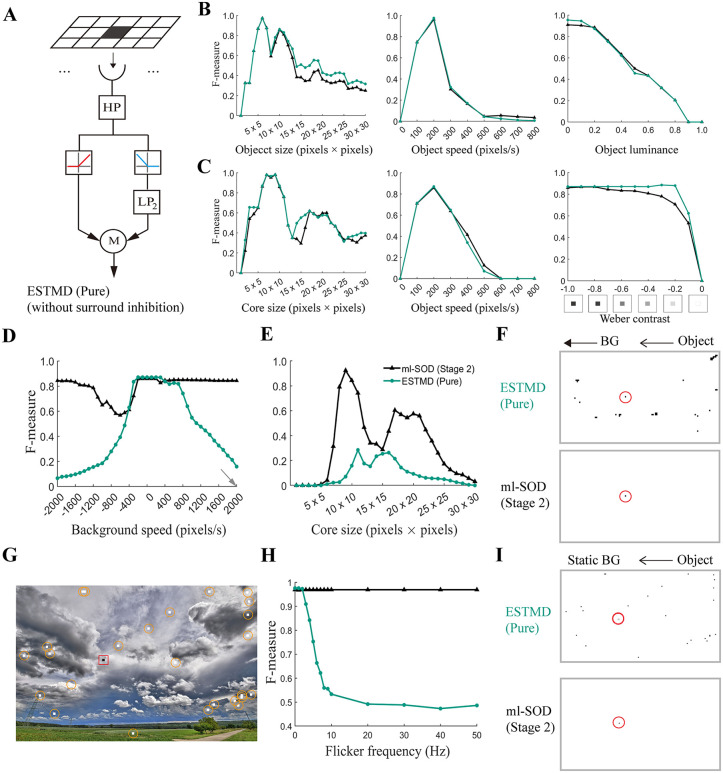
Comparison between the ml-SOD and ESTMD (pure) model. **(A)** Schematic diagram of the ESTMD (pure) model. An array of ESTMDs (signified by the parallelogram) is built to match the array size of the EMD units in the pathway of dark object detection of the ml-SOD model. The symbolic representations and parameter values in individual ESTMDs (below the parallelogram) are exactly the same as those explained in Fig 1B. **(B)** Size (left), speed (middle), and luminance (right) tuning curves of the two models under the condition of a stationary background. The stimuli include the same object as shown in the bottom left corner of Fig 4A. Unless specified in each panel, the object is 6 pixels×6 pixels in size, 0.0 in luminance, and 200 pixels/sec in speed of motion. **(C)** Same as B except that the small object is replaced with the high-contrast object, as shown in the bottom right corner of Fig 4A. Unless specified in each panel, the stimulus parameters are *d* = 10 pixels, *D* = 40 pixels, *I*_C_ = 0.0, *I*_S_ = 1.0, and *V*_object_ = 200 pixels/sec. Weber contrast is controlled by fixing *I*_S_ = 1.0 and varying the *I*_C_ in the range of 0.0‒1.0 between trials. **(D‒F)** Effects of background speed (D) and core size (E) on the detection of high-contrast objects. Unless specified in each panel, the stimulus parameter values are the same as those given in **C.** The gray arrow in D indicates *V*_background_ = +2000 pixels/sec, i.e., the background velocity used in **E.** Instantaneous response examples of the two models **(F)** (S1 Video) are from E with *d* = 10 pixels (arrows: background (BG) and object directions). (G‒I) Effect of flicker noise on the detection of a small object (6 pixels×6 pixels, 0.0 luminance, and 200 pixels/sec, marked by a red box) moving on a stationary image [26] background with 25 flickering dots inserted. The 25 dots, each with the same size as the object, are randomly distributed (marked by orange circles) and synchronously switched between black and white at a constant frequency **(G)**. Instantaneous response examples of the two models with a 50 Hz flicker frequency are displayed in **I.** Red circles mark the detected object. Data only at model stage 2 are displayed throughout. The color legend (E) is used throughout B‒E and **H.**

For simplicity, the comparison uses stimuli that include only dark objects. To ensure an unbiased comparison, we develop a model called ESTMD (pure) consisting of a 2D array of pure ESTMDs (Fig 5A). By “pure”, we mean that the ESTMDs retain multiplicative correlations between ON and delayed OFF luminance signals at the same retinotopic location but exclude all other mechanisms such as center–surround antagonism and fast depolarization and slow repolarization (FDSR) [5]. All the processing units of the retained mechanism, including high-pass and low-pass filters and half-wave rectifiers, are carefully developed to share the same parameter values with our ml-SOD model. The ESTMD (pure) model has neither stages 1 nor 3; thus, it is only compared with stage 2 of the ml-SOD model.

Under the condition of stimulus with a stationary background, the ESTMD (pure) model exhibits qualitatively similar tuning curves to those of the ml-SOD model. For example, both models show similar selectivity for object size and velocity (left and middle panels in Fig 5B) and a decreasing F-measure curve with increasing object luminance (right panel, Fig 5B). Even if the small object is replaced with a contrast-enhanced object, the similar characteristics of the tuning curve pairs between the two models are qualitatively maintained (Fig 5C). When the stimulus background is no longer static, however, the two models show fundamental differences. While the ml-SOD model performs well across all background velocities tested, the ESTMD (pure) model cannot reach an F-measure of 0.5 for background velocities less than −400 pixels/sec or greater than +1100 pixels/sec (Fig 5D).

To investigate whether object size affects the above comparison, we repeat simulations by fixing the background velocity at +2000 pixels/sec and systematically varying the object size (Fig 5E). The results show that the ml-SOD model still exhibits a higher F-measure than the ESTMD (pure) model across the entire object size range tested. Examples of instantaneous responses of the two models with an object size 10 pixels×10 pixels are shown in Fig 5F (S1 Video). Compared with the ml-SOD model, the ESTMD (pure) model results in much more noise in the detection output.

We further test the models using stimuli with flickering dots inserted in the static background (Fig 5G). The ml-SOD model demonstrates a high F-measure close to 1.0 across the entire range of flicker frequency tested, whereas the performance of the ESTMD (pure) model rapidly declines when the flicker frequency exceeds 2 Hz (Fig 5H and 5I). The robust anti-flicker property of the ml-SOD model is qualitatively similar to the flicker insensitivity exhibited by the LCs sensitive to small moving objects [3,7].

In summary, under the condition of moving background stimuli, the ml-SOD model is much more robust than the ESTMD (pure) model. Therefore, combining visual motion and luminance features gives the ml-SOD model the ability to resist interference noise elicited by background movement.

### 2.5. Replacing EMDs with three-arm detectors did not result in model performance degradation

Recent advances in understanding the fly visual system reveal that T4/T5 cells detect visual motion in a manner that can be described by a three-arm detector model, which possesses three, not two, spatially separated inputs [9–15]. It is necessary to investigate the performance of the ml-SOD model if all its EMDs are replaced with three-arm detectors (denoted as ml-SOD (three-arm)) (Methods).

**Fig 6:**
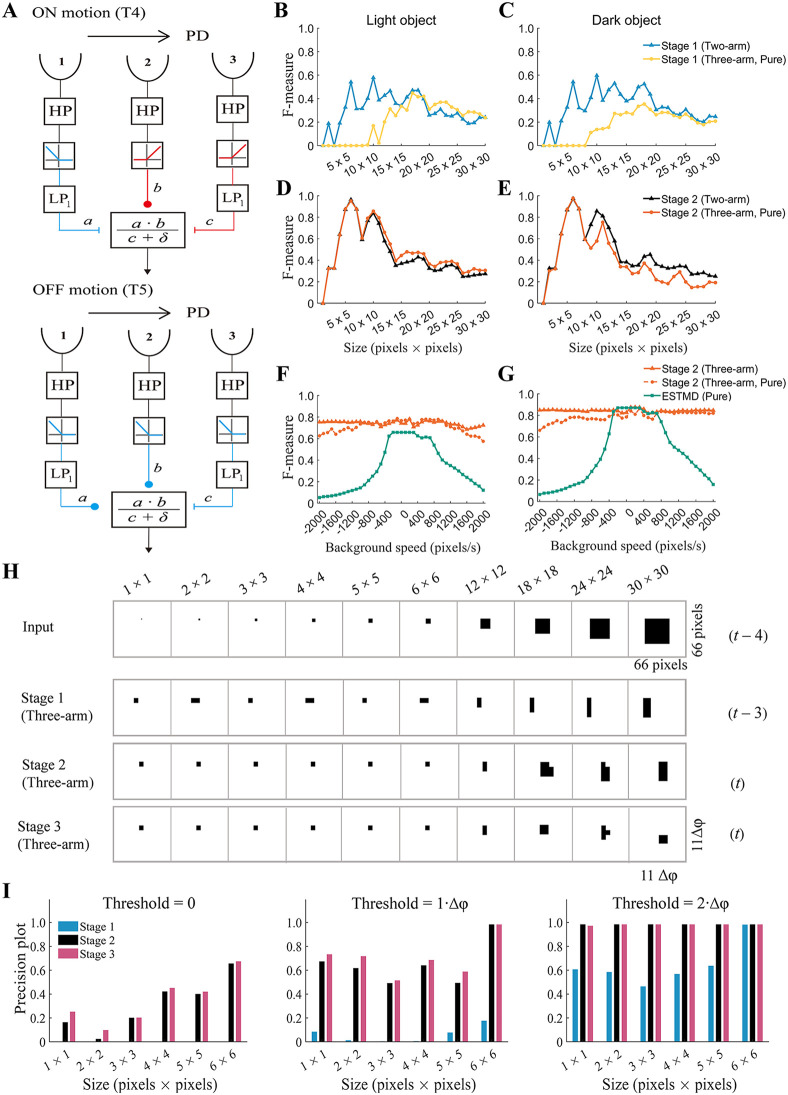
Performance of the ml-SOD model with three-arm detectors. **(A)** Schematic diagram of individual three-arm detectors. Their detailed structure is differentiated according to whether the detector belongs to the ON (upper) or OFF (lower) channel. **(B‒E)** Size tuning curves of the ml-SOD (two-arm) and ml-SOD (three-arm, pure) models with a stationary background. Data are presented vertically according to object luminance (top). The stimuli in the 1st and 2nd columns are the same as those used in Fig 2D and 2C, respectively. **(F‒G)** Comparison between the ml-SOD (three-arm) and ESTMD (pure) models under the combined condition of a moving background and a high-contrast object (*d* = 10 pixels, *D* = 40 pixels, and *V*_object_ = 200 pixels/sec). The object contrast is opposite between F (*I*_C_ = 1.0, *I*_S_ = 0.0) and G (*I*_C_ = 0.0, *I*_S_ = 1.0) (see Fig 4A for details). **(H)** Temporally aligned responses of the ml-SOD (three-arm) model at a given instant (*t* = 55). The data are from a patch of the model with a size 11Δφ×11Δφ and are displayed in the same way as in Fig 3A. **(I)** Precision plots of the ml-SOD (three-arm) model with different distance thresholds (top). The data are displayed in the same way as those in Fig 3E. The parameter *δ* is set to 0.01 for all the simulations throughout this figure. The labels “(three-arm)” and “(three-arm, pure)” represent the results with and without the use of adaptive thresholds, respectively. The data in H‒I are from the same simulations as those presented in **C.**

To ensure a fair investigation, the model retains the same structure and parameter values as those in Fig 1, except that the EMDs are replaced with three-arm detectors (Fig 6A). Since three retinotopic locations (marked as 1, 2, and 3 in Fig 6A) are needed for visual motion to be extracted, the model should combine delayed visual motion signals of the leading edge at location 3 and undelayed luminance signals of the trailing edge of opposite polarity at location 1.

By presenting the model with a light object moving over a stationary background, simulations indicate that the pathway of light object detection exhibits a lower F-measure at stage 1 than that with two-arm EMD detectors (denoted as ml-SOD (two-arm)) until the object size is larger than approximately 20×20 pixels (Fig 6B). In contrast, at stage 2, the performance of the model showed is nearly the same regardless of which type of motion detector is used (Fig 6D). When the stimulus object is replaced with a dark object, similar results are obtained in the pathway of dark object detection (Fig 6C and 6E).

Like ml-SOD (two-arm), the size selectivity of ml-SOD (three-arm) occurs mainly along the axis parallel, not orthogonal, to the direction of object motion (Fig 6H). The model can detect tiny objects smaller than the spatial resolution (∆φ = 6 pixels) of the modeled eye at each of the three stages (Fig 6H). Further re-evaluation using a precision plot reveals that its detection precision notably increases with increasing distance threshold at all three stages and reaches 1.0 at stages 2 and 3 when the distance threshold is increased to 2∆φ (Fig 6I), confirming that the ml-SOD (three-arm) model is capable of hyperacute object detection at stages 2 and 3. With respect to stage 1, ml-SOD (three-arm) is inferior to ml-SOD (two-arm) at stage 1 (Fig 6B, 6C, and 6I). These results demonstrate that visual motion detectors with two-arm EMDs are more accurate at detecting the location of tiny objects than those with three spatially separated inputs.

In the scenario of a moving background and a high-contrast object, simulations indicate that the resistance of the ml-SOD (three-arm) model to interference from background motion decreases with the parameter *δ* in the detector algorithm $\frac{a\times b}{c+\delta }$. This result is not surprising because this parameter is added to prevent division by zero and should thus be as small as possible. By setting *δ* as a small value (see the legend in Fig 6), the results, with either light or dark objects, reveal qualitatively similar performance to that of ml-SOD (two-arm) (orange dashed curve with small dots in Figs 6F and 6G versus 5D).

Since the three spatially separated inputs may make the ml-SOD (three-arm) model more susceptible to interference from background motion than the two inputs, applying an adaptive threshold to gate visual motion should be helpful for removing noise. By setting the threshold as half the maximal value of visual motion detected along the horizontal axis every time step, three-arm detectors along this axis give output only when their output exceeds the threshold. A similar operation is applied to three-arm detectors along the vertical axis. By repeating the simulations with the adaptive threshold included, the results indicate that the performance of the ml-SOD (three-arm) model is enhanced (orange solid curve with small triangles, Fig 6F and 6G). Compared with the ESTMD (pure) model, the ml-SOD (three-arm) model performs better across all the background velocities tested (Fig 6F and 6G, S1 Video).

In summary, the performance of the ml-SOD model remains qualitatively the same when its EMDs are replaced with three-arm detectors.

### 2.6. Robustness of the model to real-world video sequences

Compared with the synthetic stimuli used above, real-world scenarios are much more challenging for any small object detection model. To valuate the model performance when facing natural scene variability, we choose the RIST [33] and IR [34] datasets, which include single dark and light objects, respectively (Methods). The latter is an infrared dataset characterized by small, dim aircraft objects. The numbers of frames per video sequence of the RIST and IR datasets vary in the ranges of (751, 3073) and (399, 3000), respectively. Instead of only using selected segments as usual, we use all the frames of each sequence to ensure that the model is adequately tested.

**Fig 7:**
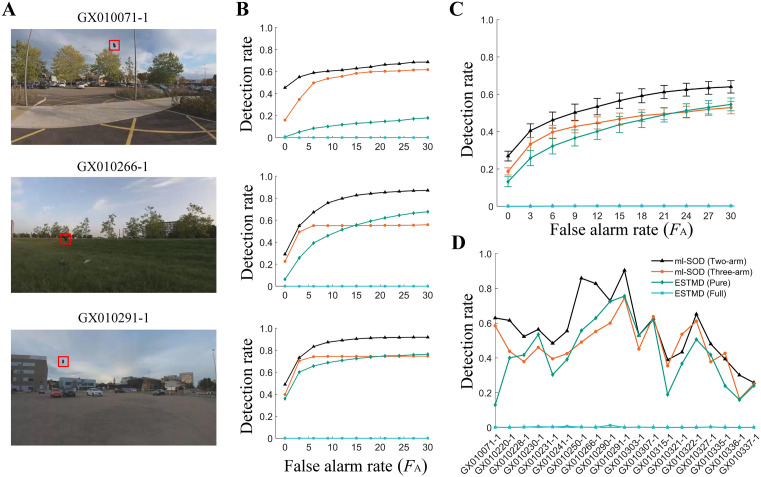
Model testing using the RIST dataset. **(A, B)** Results when three example video sequences are used. Data are presented horizontally according to sequence name (top, **A)**. Input frame examples [33], each for one sequence, are displayed in A, where red boxes mark the objects. The *D*_R_ versus *F*_A_ curves of the four models are displayed in **B. (C, D)** Results using all 19 video sequences. In addition to the curves of *D*_R_ versus *F*_A_ **(C)**, all the *D*_R_ values corresponding to *F*_A_ = 15 pixels are displayed **(D)**. ESTMD (full) is simulated by the code downloaded from https://doi.org/10.25909/21914208.v1 (located in the subfolder “IIT_Model” of the ACRA2019.zip). All the data for each ml-SOD model are based on stage 2. The time step of the input frames is set to Δ*h* = 1/*f*_video_ = 4.2 ms. The ml-SOD (three-arm) model includes the adaptive threshold proposed in section 2.5. The color legend in D is used throughout.

We first test the pathway of dark object detection by feeding each of the 19 RIST sequences to the ml-SOD model (two-arm) (Fig 7). For comparison, the testing is repeated with three other models: ml-SOD (three-arm), ESTMD (pure), and ESTMD (full). The ESTMD (full) model is a full version of ESTMD, which is just the insect-inspired tracker (IIT) with its tracking subsystem removed [35]. ESTMD (full) can be viewed as an extension of the ESTMD (pure), because the former includes mechanisms such as center–surround antagonism and fast adaptation. We simulate the ESTMD (full) using the code published in the literature [36], of which only the portion corresponding to early visual processing and target-matched filtering ESTMD stage is intercepted. The code is used without any alterations except that the additive operation on two parallel pathways separately for dark and light object detection is removed. Only the pathway for dark object detection in their code is tested to ensure a fair comparison.

The results are evaluated by averaging the detection rate (*D*_R_) and false alarm rate (*F*_A_) values across all the input sequences (Methods). The metrics, which are based on either three single sequences (Fig 7B) or all the sequences (Fig 7C), indicate that the ml-SOD (two-arm) model exhibits the best performance and that the ml-SOD (three-arm) model performs better than the ESTMD (pure) model. The ESTMD (full) model shows the worst performance, with detection rates close to zero. To check the effect of sequence variability on object detection, the *D*_R_ metric under the condition of an intermediate false alarm rate (*F*_A_ = 15 pixels) is displayed in Fig 7D.

**Fig 8:**
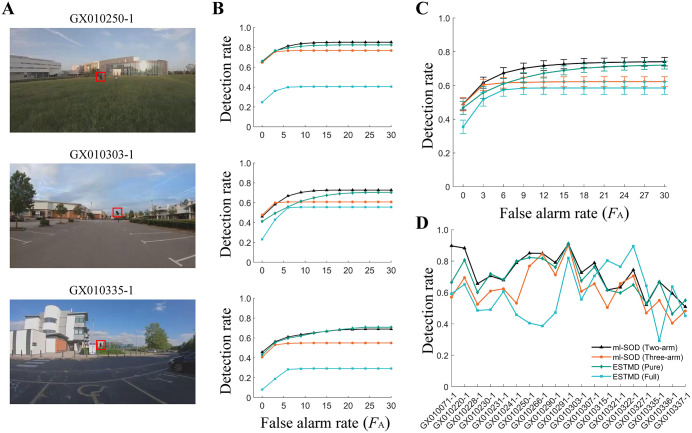
Model testing using the downsampled RIST dataset. **(A‒D)** Panels A, B, C, and D are the same as panels A, B, C, and D in Fig 7, respectively, except for two differences. First, each frame (denoted as ‘img0’) is downsampled using MATLAB’s built-in function ‘imresize(img0, 1/3, ‘bilinear’)’ before being fed into the models. The corresponding ground truth data are downsampled by retaining one pixel at three-pixel intervals along both dimensions, ensuring dimensional consistency with the model output. Second, the three example sequences in A and B [33] are different from those in Fig 7. The color legend in D is used throughout.

The extremely low detection rates of the ESTMD (full) model may occur because the RIST objects are larger than the preferred object size of the model and are thus suppressed by its center–surround antagonism. Even for the ml-SOD models, the object sizes of the RIST dataset (3×3 pixels to 15×15 pixels) are much larger than the spatial resolution of the modeled eye (1×1 pixels). Note that no preprocessing is applied to the dataset (Methods). Increasing the parameter *τ* should increase the *D*_R_ because the model prefers an object size *S* = *V* x *τ*. Nevertheless, we adopt another strategy, i.e., downsampling the input frames to improve the detection rate, as downsampling can increase the detection speed. The results indicate that this strategy generally increases the *D*_R_ values of all four models, confirming that the parameter values of all these models prefer smaller objects than the original object sizes in the RIST dataset (Fig 8).

The two ml-SOD models are outperformed by the ESTMD (full) when facing a few sequences, such as GX010315–1, GX010321–1, GX010322–1 and GX010327–1 (Fig 8D). In addition to challenges (e.g., spots and speckles in the moving background that have similar spatiotemporal properties to the object) common to all the models tested, the ml-SOD models are further challenged by elongated building edges, elongated zebra crossings, rods, etc. This phenomenon arises because the ml-SOD models cannot suppress responses to features extended along the axis orthogonal to the target motion. This characteristic arises because the model is not equipped with the center–surround antagonism that is considered an essential mechanism of the ESTMD (full) model. Moreover, the approximate zero false alarm rate (*F*_A_ = 0) of the ESTMD (pure) model has a similar *D*_R_ to that of the ml-SOD models (Fig 8C). This result may occur because the local contrast of objects in the RIST dataset is not adequately high.

**Fig 9:**
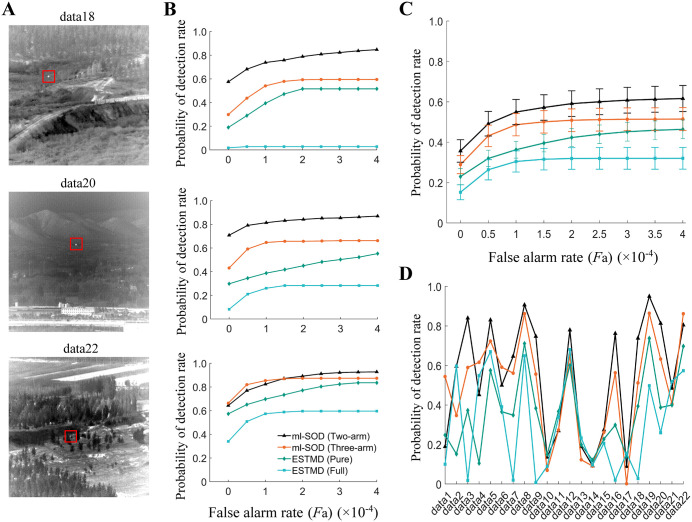
Model testing using the IR dataset. **(A, B)** Results when three example video sequences are used. Data are presented horizontally according to sequence name (top, **A)**. Input frame examples [34], each for one sequence, are displayed in A, where red boxes mark the objects. The *P*_d_ versus *F*_a_ curves of the four models are displayed in **B. (C, D)** Results using all 22 video sequences. In addition to the curves of *P*_d_ versus *F*_a_ **(C)**, all the *P*_d_ values corresponding to *F*_a_ = 1 × 10^‒4^ are displayed **(D)**. All the data for each ml-SOD model are based on stage 2. The time step of the input frames is set to Δ*h* = 1/*f*_video_ = 10 ms. The ml-SOD (three-arm) model includes the adaptive threshold proposed in section 2.5. Only the pathway for the light object detection of the ESTMD (full) model is tested to ensure a fair comparison. The color legend presented in the bottom panel in B is used throughout.

Using each of the 22 video sequences in the IR dataset, we next test the pathway of light object detection. Simulation results, which are based on either three single sequences (Fig 9A and 9B) or all the sequences (Fig 9C), reveal that the best performer is still the ml-SOD model (two-arm), followed by the ml-SOD (three-arm), ESTMD (pure) and ESTMD (full) models. However, the ml-SOD model is challenged when faced with certain sequences, such as data1, data10, data11, data13‒15, and data17 (Fig 9D). The underlying reasons vary depending on the sequence. For example, in some sequences (e.g., data1), the small object barely changes its position within the visual field for many frames, preventing the model from extracting its motion feature. In other sequences (e.g., data10 and data17), the local contrast of small objects is too low to be distinguished from the background, leading to a low detection rate.

**Fig 10:**
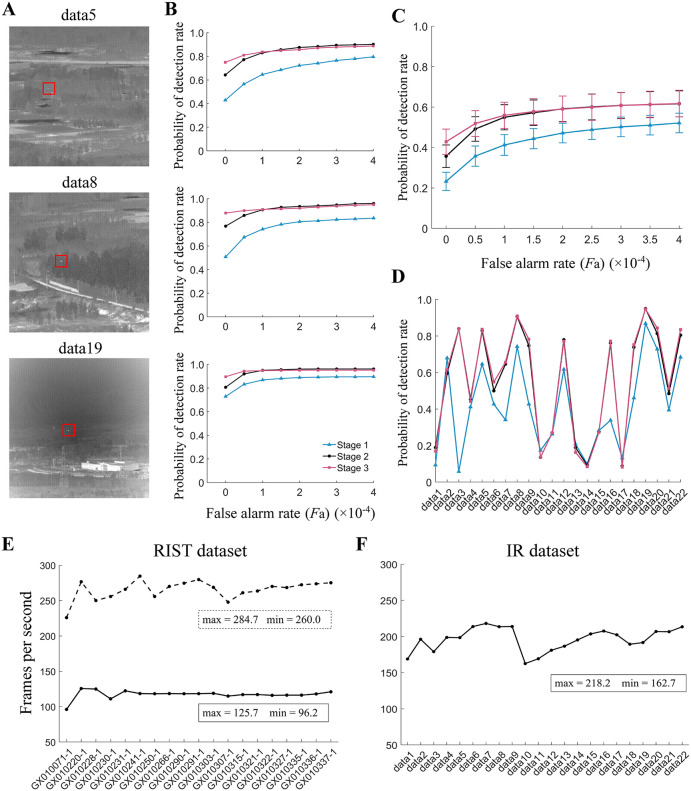
Comparison between performances at three model stages with the IR dataset. **(A‒D)** Panels A, B, C, and D are the same as panels A, B, C, and D in Fig 9, respectively, except for two differences. First, the three example sequences in A and B [34] are different from those in Fig 9. Second, only the ml-SOD (two-arm) model is tested in three stages. The color legend (bottom panel in B) is used throughout. **(E, F)** Detection speed of the ml-SOD (two-arm) model with its stage 3 removed. The solid and dashed curves represent the results without and with downsampling preprocessing to video frames of the corresponding dataset (top), respectively. The maximum and minimum fps values are indicated (the boxes beside the curves).

Given that at a low false alarm rate around *F*_A_ = 0, the ml-SOD model with either two- or three-arm detectors outperforms the ESTMD-based model with both datasets (Figs 7‒9), we wonder to what extent the utilization of visual motion features can help increase the detection rate. We then examine the *P*_d_ metric at all three stages of the ml-SOD (two-arm) model. The results indicate that even at stage 1, i.e., the stage for extracting visual motion, the model is comparable to the ESTMD (pure) model (cyan curve in Figs 10C versus green curve in 9C), confirming the critical role of the visual motion feature.

The ml-SOD model features low complexity. Although we have not put much effort into optimizing the speed on purpose, the processing speeds at stage 2 reach frames per second (fps) values greater than 90 and 160 for the RIST and IR datasets, respectively (Fig 10E and 10F). In the case of the downsampled RIST dataset, the fps at stage 2 increases to over 260 (dashed curve, Fig 10E).

Taken together, compared with the ESTMD-based model, the ml-SOD model is more robust and efficient for small moving object detection tasks in real-world video sequences. When facing natural scene variability, the ml-SOD model with two-arm EMDs performs better than that with three-arm detectors. The major reason may be that the three spatially separated inputs, especially the input that exerts a divisive inhibiting effect, are more susceptible to interference from a moving background than the two-input model.

## 3. Discussion

### 3.1. Predictions of variants of the feature combination model

**Fig 11:**
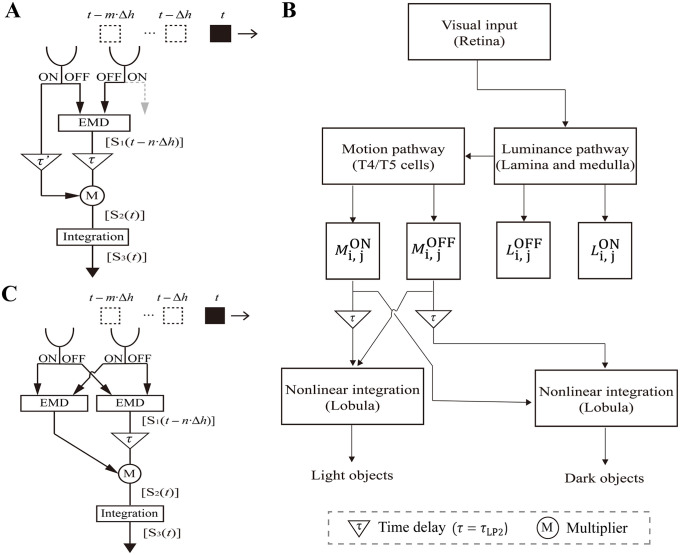
Predicted variants of the feature combination model. **(A)** Schematic diagram of the calculation structure of the first variant at a pair of adjacent retinotopic locations. The structure is the same as that in Fig 2B1, except for one additional signal delay *τ*′ in the luminance pathway. **(B‒C)** Schematic diagram of the second variant. This variant connectivity (B) is the same as that in Fig 1A except that the features combined are both visual motion features with opposite polarity. The calculation at a pair of adjacent retinotopic locations correlates the visual motion features of both the leading and trailing edges of the small object **(C)**. The pathway of dark object detection is displayed in A and **C.**

From an algorithmic point of view, the ml-SOD model predicts that two possible variants of network connectivity exist. The first variant proposes that the temporal delay *τ*_LP2_ may occur not only in the visual motion but also in the luminance signaling pathway, as long as the two signals, which separately encode the leading and trailing edges of the object, can finally meet at the same retinotopic location (Fig 11A).

Another variant proposes that small object detection should even be complemented with visual motion signals alone (Fig 11B). This variant detects small moving objects by combining visual motion signals with opposite polarity at adjacent retinotopic locations, which can be called mm-SOD, and should be equivalent in detection effectiveness to the original version of the ml-SOD model. For example, consider the pathway for dark object detection. The mm-SOD variant retinotopically multiplies the delayed visual motion signal in the OFF channel (denoted as matrix $M_{\text{i},\text{ j}}^{OFF}$) by the undelayed visual motion signal in the ON channel (denoted as matrix $M_{\text{i},\text{ j}}^{ON}$), yielding the output of stage 2 as $M_{\text{i},\text{ j}}^{OFF}\times M_{\text{i},\text{ j}}^{ON}$ (Fig 11C).

From the point of view of the fly visual system, however, the network connectivity of the ml-SOD model and its variants that is anatomically constrained remains unknown. Considering that a large majority of outputs from the medulla and the lobula plate converge to the lobula in *Drosophila* [37,38], we predict the neural substrate of stage 3 of the ml-SOD model corresponds to the lobula columnar (LC) cells sensitive to small objects. Future studies, especially on the functional manipulation of specific visual circuits through improved genetic tools in *Drosophila*, are expected to provide insights into these predictions. For example, the critical role of visual motion proposed by our model can be experimentally validated using motion-blind flies whose synaptic outputs from T4/T5 cells are blocked [39]. According to the ml-SOD model, motion-blind flies fail to detect small objects moving independently.

### 3.2. Comparison with bioinspired models

Among previous models inspired by the insect visual system, only the cascaded EMD-ESTMD model [16] involves visual motion. Although the cascaded model seems to be similar in form to one of our model variants (Fig 11B and 11C), the two models are essentially different. The cascaded model is incomplete, as its direction selectivity involves only one of the four cardinal directions. It is unclear how the cascaded model detects a small object that frequently changes direction. In contrast, our model and its variants first capture the visual motion features of the object, regardless of its direction. Our model predicts that the feature combination-based LC cells should be motion-selective yet nondirectional.

The ESTMD model [2,5,6] and ESTMD-based models [19,20,40] usually incorporate additional mechanisms for generating size selectivity along the axis orthogonal to small object motion. In contrast, we focus on developing and dissecting a minimal model in the present study, although our model does not exclude the possibility of such strong center–surround antagonism.

Few insect-inspired models have been shown to be functional under the condition of real-world datasets of small object detection. Compared with our model, which is validated with two complete datasets, other ESTMD-based models are tested with no more than one-third of the video sequences of one dataset [20,40]. One of these models uses the RIST dataset, similar to our model [40]. Testing using 6 of the 19 RIST sequences reveals that the aforementioned model exhibits a very low detection rate at a false alarm rate of zero (Fig 16 in [40]). By comparison, our ml-SOD model demonstrates a remarkable increase in the zero false alarm rate even when tested across all the RIST sequences (Figs 7C and 8C), indicating that the combination of visual motion and luminance features is effective and efficient for small moving object detection.

Another type of model is inspired by the function of the retinal magnocellular pathway in the human visual system [41,42]. Unlike our ml-SOD model, which uses EMDs to estimate visual motion, this type of model extracts the motion strength of moving objects with spatiotemporal filter banks. This type of model does not work independently but instead cooperates with deep convolutional neural networks by enhancing the areas of moving objects and thereby improving the performance of deep neural networks on tiny drone detection tasks [41,42]. This finding is in contrast to the ml-SOD model, which can detect small objects independently without the need for any learning or training.

### 3.3. Limitations of the study

The major shortcoming of the model lies in its inability to detect static objects. Thus, a small object with intermittent motion is not detected once it stops moving, although the detection is restored as soon as the object movement resumes. This failure occurs due to a lack of visual motion, which is key for the ml-SOD model. This problem may be overcome in the future by adding a parallel tracking mechanism to the model. This mechanism can accumulate historical responses to targets moving on continuous trajectories. Such response characteristics have been identified in a specific type of dragonfly STMD neuron named CSTMD1 [43], which inspires a small target tracker [35,44]. Whether similar neurons sensitive to the movement history of targets exist in the fly visual system is not yet clear.

Any object appears as a small target when viewed at a far distance. Although the detection of objects with variable sizes caused by changes in depth is not addressed here, we suppose that an adaptive model comprising multiple parallel modules is needed. Such a model should be capable of switching between the detection modules for normal objects [24,25] and tiny objects (e.g., the ml-SOD model) based on the input scenario.

In summary, a minimal model is developed to combine the two features that small moving objects have: visual motion and luminance. By dissecting the model performance with tiny objects smaller than the spatial resolution of the modeled eye, the model is demonstrated to be capable of hyperacute object detection. Compared with existing models that rely solely on luminance features, the model shows enhanced resistance to interference from moving backgrounds, even when tested with full-length real-world video sequences with natural scene variability. These characteristics are independent of whether the motion detectors have two or three spatially separated inputs.

## 4. Methods

### 4.1. Visual stimuli

Two types of stimuli were used in the simulations. The first was artificial stimuli, which were synthesized by superimposing an independently moving square object onto an image background. The size, velocity, and luminance of the object and the background velocity were adjustable. The object luminance was simulated as a dimensionless quantity ranging from 0.0 (dark) to 1.0 (light). The image was a natural scene taken from a weather dataset with a resolution of 1024×675 pixels [26]. Only the green channel of the image, which was normalized to the range (0, 1), was used as input. To model the low-resolution vision of flying insects, each frame was first blurred using a Gaussian low-pass filter with a size of 12×12 pixels and a standard deviation of σ=3.5 pixels (using the MATLAB function ‘fspecial’) and then downsampled by retaining one pixel at six-pixel intervals along both dimensions. Preprocessing enabled the model to simulate the optical properties of the fly compound eye with a photoreceptor acceptance angle of $\text{2}\sqrt{\text{2}ln\text{2}}\sigma =\text{8}.\text{2}$ pixels (full width at half maximum) and an interommatidial angle of 6 pixels. Therefore, the spatial resolution of the modeled eye was ∆φ = 6 pixels when the input was synthetic stimuli.

The second type was real-world video sequences in the RIST dataset [33] and the IR dataset [34], both of which were taken in various complex scenes using nonstationary cameras. For both datasets, only the green channel of the image, which was normalized to the range (0, 1), was used as the luminance input. No additional processing mimicking light adaptation in the retina was applied to the input, as the two datasets were not high-dynamic-range images.

The RIST dataset contained 19 sequences with a resolution of 480×270 pixels and a frame rate of *f*_video_ = 240 Hz. Each sequence contained a dark object, ranging in size from 3 pixels×3 pixels to 15 pixels×15 pixels. An object moved independently in dynamic environments, whose background varied among the sky, buildings, trees, grasslands, and shrubs under sunny or cloudy lighting conditions. The IR dataset was taken using infrared sensors in complex outdoor environments, and it contained 22 sequences with a resolution of 256×256 pixels and a frame rate of *f*_video_ = 100 Hz. Each sequence contained a single dim small aircraft object, except for data2 and data4, which contained two objects. The infrared object occupied approximately 0.12% of the frame size [45]. Unless otherwise specified, no preprocessing (i.e., Gaussian blur or downsampling) was applied to the second type of stimulus. Therefore, the spatial resolution of the modeled eye was ∆φ = 1 pixel when the input was real-world video sequences.

The stimulus images were sequentially fed to the model with a time interval Δ*h*, which was set to Δ*h* = 10 ms and Δ*h* = 1/*f*_video_ for the first and second types of stimuli, respectively.

### 4.2. Extracting luminance and nondirectional visual motion signals

At each retinotopic location, the model began with a first-order high-pass filter with a time constant of *τ*_HP_ = 30 ms, whose output was then divided into the ON and OFF channels using the ON and OFF half-wave rectifiers, respectively (Fig 1B). Both rectifiers had a cutoff set to 0, through which a pair of ON and OFF luminance signals were extracted at the retinotopic location.

Visual motion was estimated using arrays of two types of EMDs. The first type of EMD had two spatially separated input channels or arms [8,46]. Each retinotopic location was equipped with two EMDs, which were each responsible for horizontal and vertical motion detection. Individual EMDs detected directional motion via parallel ON and OFF channels, both of which consisted of two symmetric half-detectors with a first-order low-pass filter (*τ*_LP1_ = 50 ms) (Fig 1B). The subtraction between outputs of the two symmetric half-detectors yielded directional motion signals with opposite signs, representing the two horizontal (or vertical) directions. By adding a full-wave rectifier to the EMD output and summing the rectified signals across the horizontal and vertical axes at each retinotopic location, nondirectional visual motion signals were finally extracted.

The model was investigated by replacing two-arm EMDs with three-arm EMDs, whose neural implementation was evidenced by recent advances [9–15]. Individual three-arm detectors were characterized by three rather than two spatially separated and colinear input channels. Central position 2 provided fast input, whereas two flanking inputs were delayed using a first-order low-pass filter (*τ*_LP1_ = 50 ms) (Fig 6A). The top panel of Fig 6A shows a T4 cell model with a rightward preferred direction. We switched the two arms at positions 1 and 3 to simulate a T4 cell tuned to leftward motion. The bottom panel of Fig 6A shows a T5 cell model with a rightward preferred direction. We switched the two arms at positions 1 and 3 to simulate a T5 cell tuned to leftward motion.

Let *a*, *b*, and *c* be the inputs to the three arms; the output of the three-arm detectors was calculated as $\frac{a\times b}{c}$ (Fig 6A). To avoid division by zero in the visual motion calculation, a constant *δ* was added to the denominator, i.e., $\frac{a\times b}{c+\delta }$. We set *δ* = 0.05 unless otherwise specified. Given that each retinotopic location was equipped with four three-arm detectors yielding positive outputs, each for one of the four cardinal directions, the final directional motion signals were determined by the two detectors with a greater output magnitude along either the horizontal or vertical axis. Nondirectional visual motion signals at the retinotopic location were ultimately extracted by summing the outputs of the two detectors.

### 4.3. Lobula modules for signal integration

The model consisted of two parallel and feedforward pathways, which separately detected dark and light objects (Fig 1A). Each pathway ended with a lobula module, which was assumed to spatiotemporally integrate signals of feature combination. Through spatial integration, individual lobula units aggregated inputs from their receptive fields. Temporal integration was attributed to the dynamic system characteristics of the lobula units obeying ordinary differential equations [24].

The membrane potential of individual lobula units was described by integrate-and-nonspiking dynamics, with an integration time step of 1 ms:


$$\tau_{\text{m}}\frac{dV_{i,\;j}(t)}{dt}=-V_{i,\;j}(t)+E_{\text{L}}+RI_{i,\;j}^{syn}(t)$$ (1)

where $V_{i,\;j}(t)$ is the membrane potential matrix of the lobula module; *τ*_m_ (5 ms) and *E*_L_ (–50 mV) are the membrane time constant and the resting membrane potential, respectively; *R* is the total membrane resistance, which was set to a dimensionless parameter (*R* = 1) for simplicity; and $I_{i,\;j}^{syn}(t)$ is the input current matrix to the lobula module that was modeled as follows:


$$I_{i,\;j}^{syn}(t)=w_{\text{ext}}g_{i,\;j}(t)(E_{\text{ext}}-V(t))$$ (2)

where $g_{i,\;j}(t)$ and $w_{\text{ext}}$= 0.1 are the excitatory conductance matrix and the synaptic weight of the input, respectively, and $E_{\text{ext}}=\text{0}$ is the reversal potential of the excitatory synaptic current.

To obtain the final output of the lobula module, the membrane potentials were mapped to a range (0, 1) using the sigmoid activation function $\text{1}/\left(\text{1}+e^{\left(\theta -V(t)\right)/\beta }\right)$, in which *θ* = ‒40 mV and *β* = 0.5 are the half-activation voltage and the steepness parameter, respectively.

### 4.4. Small object detection evaluation

For most simulations, the model was evaluated not only at the last stage (i.e., stage 3) but also at each of the intermediate stages along the signal processing flow (Fig 1). Before any evaluation, the output matrix at a given stage was segmented into foreground and background pixels every interval Δ*h*. The segmentation threshold was set to 50% of the maximum element value of the matrix. The degree to which the binary images were consistent with the ground truth was then evaluated using stimulus-dependent metrics.

For the first type of stimulus, both the location and the boundary of the small object were considered the ground truth. Therefore, the F-measure was used, which accounted for both precision and recall [47]:


$$F-measure={\left(\frac{{precision}^{-\text{1}}+{recall}^{-\text{1}}}{\text{2}}\right)}^{-\text{1}}=\frac{\text{2}TP}{\left(\text{2}TP+FP+FN\right)}$$ (3)

where *precision* = *TP*/(*TP* + *FP*) and *recall* = *TP*/(*TP* + *FN*) (see the Methods in [24] for details); *TP* (i.e., true positives) and *FP* (i.e., false positives) are the numbers of foreground pixels correctly and incorrectly detected, respectively; and *FN* (i.e., false negatives) is the number of background pixels incorrectly detected. The F-measure value ranged from 0 to 1, indicating that the model performance ranged from the worst to the best. We calculated average F-measures in this study. Specifically, *TP*, *FP*, and *FN*, which were calculated for each binary image, were separately accumulated across the stimulus presentation. The average F-measures were finally obtained by substituting the accumulated *TP*, *FP*, and *FN* into formula (3). Note that no Gaussian blur was required for the ground truth. However, the ground truth needed to be downsampled by retaining one pixel at six-pixel intervals along both dimensions to maintain consistency with the sizes of the downsampled input frames.

The performance for detecting tiny objects smaller than the spatial resolution of the modeled eye was evaluated using a precision plot. The precision metric was defined as above, i.e., *precision*′ = *TP*′/(*TP*′ + *FP*′), except that the true positives (*TP*′) and the false positives (*FP*′) represent the numbers of foreground pixels correctly and incorrectly detected under the condition of a given distance threshold, respectively. Specifically, *TP*′ (*FP*′) is the number of detected foreground pixels whose Euclidean distance from the ground truth location is not greater (greater) than the threshold. We only calculated the average *precision*′ in this study. Specifically, *TP*′ and *FP*′, which were calculated every interval Δ*h*, were separately accumulated across the stimulus presentation. The accumulated values were substituted into the *precision*′ formula to obtain the average.

For the second type of stimulus, i.e., real-world video sequences, only the central locations of small objects were available in the ground truth. Since the F-measure was no longer considered suitable, we adopted dataset-dependent metrics proposed by previous studies using a specific dataset. The metric pair of detection rate (*D*_R_) and false alarm rate (*F*_A_) [40] was used to evaluate model performance with the RIST dataset [33]. For the single object detection task, the metric pair was defined as follows:


$$D_{\text{R}}=\frac{N_{\text{TD}}}{N_{\text{Frame}}},\;F_{\text{A}}=\frac{N_{\text{FD}}}{N_{\text{Frame}}}$$ (4)

where *N*_Frame_ is the number of input frames and *N*_TD_ and *N*_FD_ are the numbers of true and false detections accumulated across all the input frames, respectively. The true and false detections were determined on a pixel-by-pixel basis. A detected pixel was determined to be true if its distance to the ground truth was not greater than 5 pixels; otherwise, it was considered falsely detected.

For simulations with the IR dataset [34], we used the metric pair of probability of detection rate (*P*_d_) and false alarm rate (*F*_a_) [48]. For the single object detection task, the metric pair was defined as follows:


$$P_{\text{d}}=\frac{N_{\text{TD}}}{N_{\text{Frame}}},\;F_{\text{a}}=\frac{N_{\text{FD}}}{N_{\text{P}}}$$ (5)

where *N*_P_ is the number of total pixels in a single frame of the input video. Note that all the frame images in the IR dataset were the same size. The metric *P*_d_ is the same as *D*_R_ in Eq (4) except for its name. *F*_a_ is the same metric as *F*_A_ in Eq (4) except for its denominator.

### 4.5. Simulations

The model was simulated in MATLAB and run on a laptop with a 12th Gen Intel(R) Core(TM) i9-12900H processor (2.50 GHz). Each input image fed to the model had a presentation time of Δ*h* (i.e., the step of individual EMD units), which was set to Δ*h* = 10 ms and Δ*h* = 1/*f*_video_ for the first and second types of stimuli, respectively. All the neuron units in the lobula modules downstream of the EMD arrays were numerically integrated with an integration step of 1.0 ms using the fourth-order Runge–Kutta method.

## Supporting information

S1 Video Time course of outputs of three models (indicated at the top) to the input with a small high-contrast object moving over an image [26] background. Shown at 0.25 **×** actual speed.
(MP4)
